# Adiponectin Provides Additional Information to Conventional Cardiovascular Risk Factors for Assessing the Risk of Atherosclerosis in Both Genders

**DOI:** 10.1371/journal.pone.0075535

**Published:** 2013-10-08

**Authors:** Jin-Ha Yoon, Sung-Kyung Kim, Ho-June Choi, Soo-In Choi, So-Youn Cha, Sang-Baek Koh, Hee-Taik Kang, Song Vogue Ahn

**Affiliations:** 1 Institute of Occupational and Environmental Medicine, Wonju College of Medicine, Yonsei University, Seoul, Korea; 2 Department of Preventive Medicine, Wonju College of Medicine, Yonsei University, Seoul, Korea; 3 Institute of Genomic Cohort, Wonju College of Medicine, Yonsei University, Seoul, Korea; 4 Graduate School of Public Health, Yonsei University, Seoul, Korea; 5 Department of Family Medicine, Yonsei University College of Medicine, Gangnam Severance Hospital, Seoul, Korea; 6 Department of Medicine, Graduate School, Yonsei University, Seoul, Korea; University of Leicester, United Kingdom

## Abstract

**Background:**

This study evaluated the relation between adiponectin and atherosclerosis in both genders, and investigated whether adiponectin provides useful additional information for assessing the risk of atherosclerosis.

**Methods:**

We measured serum adiponectin levels and other cardiovascular risk factors in 1033 subjects (454 men, 579 women) from the Korean Genomic Rural Cohort study. Carotid intima–media-thickness (CIMT) was used as measure of atherosclerosis. Odds ratios (ORs) with 95% confidence intervals (95% CI) were calculated using multiple logistic regression, and receiver operating characteristic curves (ROC), the category-free net reclassification improvement (NRI) and integrated discrimination improvement (IDI) were calculated.

**Results:**

After adjustment for conventional cardiovascular risk factors, such as age, waist circumference, smoking history, low-density and high-density lipoprotein cholesterol, triglycerides, systolic blood pressure and insulin resistance, the ORs (95%CI) of the third tertile adiponectin group were 0.42 (0.25–0.72) in men and 0.47 (0.29–0.75) in women. The area under the curve (AUC) on the ROC analysis increased significantly by 0.025 in men and 0.022 in women when adiponectin was added to the logistic model of conventional cardiovascular risk factors (AUC in men: 0.655 to 0.680, p = 0.038; AUC in women: 0.654 to 0.676, p = 0.041). The NRI was 0.32 (95%CI: 0.13–0.50, p<0.001), and the IDI was 0.03 (95%CI: 0.01–0.04, p<0.001) for men. For women, the category-free NRI was 0.18 (95%CI: 0.02–0.34, p = 0.031) and the IDI was 0.003 (95%CI: −0.002–0.008, p = 0.189).

**Conclusion:**

Adiponectin and atherosclerosis were significantly related in both genders, and these relationships were independent of conventional cardiovascular risk factors. Furthermore, adiponectin provided additional information to conventional cardiovascular risk factors regarding the risk of atherosclerosis.

## Introduction

Adipose tissue, beyond the concept of functioning as a passive energy reservoir, has been studied as an active endocrine organ linked to atherosclerosis [1]. Adipose tissue synthesizes and secretes various adipokines, such as adiponectin and leptin [2]. Adiponectin is likely to play a protective role against atherosclerosis and cardiovascular risk [3]. The expression of adiponectin is inversely correlated with obesity, insulin resistance [4], and development of early atherosclerosis [5]. Because adiponectin exerts protective effects on the cardiovascular system, it could also be highlighted as a therapeutic target molecule for preventing atherosclerosis and atherosclerotic events [6]. Furthermore, we have previously reported that adiponectin has an inverse correlation with carotid intima–media thickness in Korean subjects, after adjustment for gender [7].

**Table 1:** Anthropometric and metabolic characteristics of study population.

	Men (n = 454)	Women (n = 579)	P value
Age, y	57.17±7.16	55.40±7.29	<.001
Waist circumference, cm	87.10±7.26	82.92±8.63	<.001
Body mass index	24.73±3.73	24.38±3.46	0.066
Smoking history, n (%)			<.001
current smoker	169 (37.22)	20 (4.41)	
ex-smoker	137 (30.18)	4 (0.88)	
non-smoker	148 (32.60)	555 (122.25)	
SBP, mmHg	128.57±15.86	130.03±16.32	0.178
DBP, mmHg	82.95±10.99	81.53±10.50	0.017
FBG, mg/dL	95.25±18.30	91.12±16.43	<.001
FBI, µIU/mL	6.90 (5.53–8.80)	7.80 (6.35–10.00)	<.001
HOMA-IR	1.60 (1.24–2.10)	1.74 (1.34–2.26)	0.003
Total cholesterol, mg/dL	201.43±36.98	211.28±40.28	<.001
Triglycerides, mg/dL	134.50 (95.00–201.00)	118.00 (85.50–169.50)	<.001
HDL-C, mg/dL	46.00 (39.00–54.00)	47.00 (41.00–55.00)	0.005
LDL-C, mg/dL	116.40±31.03	124.84±33.99	<.001
Adiponectin, mg/L	7.82 (5.63–11.05)	11.53 (8.41–14.86)	<.001
CIMT, mm	0.85 (0.72–1.00)	0.84 (0.72–0.96)	0.056
% of high CIMT (>0.9 mm), n (%)	197 (43.39)	227 (39.21)	0.196

SBP, systolic blood pressure; DBP, diastolic blood pressure; FBG, fasting blood glucose; FBI, fasting blood insulin;, HOMA-IR, Homeostasis model assessment-insulin resistance; HDL-C, high-density lipoprotein cholesterol; LDL-C, low-density lipoprotein cholesterol; CIMT, carotid intima media thickness.

However, some studies reported that adiponectin was a useful marker of atherosclerosis in men, but not in women [8]. Furthermore, the Framingham Offspring Study also revealed that adiponectin is an independent risk factor for coronary heart disease in men, but this association was attenuated and lost its significance in women after adjustment for conventional risk factors for cardiovascular diseases, including obesity parameters [9]. The MONICA/KORA Augsburg study indicated that adiponectin is insufficient for predicting the prognosis of atherosclerosis after adjustment for conventional cardiovascular risk factors [10]. However, it is unknown whether these results were indicative of the exact relation between adiponectin and atherosclerosis in each gender because the MONICA/KORA Augsburg study did not use stratified analyses according to gender. Therefore, we undertook this investigation to elucidate the role of adiponectin for each gender separately.

**Table 2:** Basic characteristics according to tertile increment of adiponectin based on each genders.

men (n = 454)	1^st^ tertile ≤6.25	2^nd^ tertile >6.25	3^rd^ tertile >9.75	P value
Age, y	55.49±6.73	57.16±7.36	58.89±7.03	<.001
Waist circumference, cm	88.08±7.24	87.80±6.95	85.42±7.35	0.002
Body mass index	24.81±2.92	24.58±4.24	24.80±3.92	0.367
Smoking history, n (%)				0.108
current smoker	66 (43.42)	49 (32.23)	51 (33.55)	
ex-smoker	48 (31.57)	48 (31.58)	41 (26.97)	
non-smoker	38 (25.00)	55 (36.18)	58 (38.16)	
SBP, mmHg	129.50±17.38	127.99±15.10	128.21±15.06	0.919
DBP, mmHg	83.31±11.86	83.01±11.31	82.53±9.75	0.826
FBG, mg/dL	98.92±21.56	95.28±17.29	91.51±14.73	<.001
FBI, µIU/mL	7.75 (6.20–9.65)	6.75 (5.57–8.57)	6.2 (5.23–8.00)	<.001
HOMA-IR	1.89 (1.43–2.45)	1.60 (1.29–2.04)	1.41 (1.14–1.73)	<.001
Total cholesterol, mg/dL	203.06±34.98	198.67±33.12	202.49±38.58	0.507
Triglyceride, mg/dL	175.00 (117.00–240.75)	130.50 (95.00–190.25)	114.50 (82.00–172.50)	<.001
HDL-C, mg/dL	43.00 (37.00–50.25)	45.00 (39.00–52.25)	49.50 (42.00–58.00)	<.001
LDL-C, mg/dL	117.79±31.72	115.63±29.39	115.77±32.08	0.795
CIMT, mm	0.91 (0.76–1.04)	0.84 (0.72–1.00)	0.84 (0.68–0.99)	0.022
% of high CIMT (>0.9 mm), n (%)	78 (51.31)	62 (40.79)	57 (37.50)	0.047*
**women (n = 579)**	**1^st^ tertile ≤9.45**	**2^nd^ tertile >9.45**	**3^rd^ tertile >13.40**	
Age, y	53.98±6.86	55.44±7.54	56.77±7.23	0.001
Waist circumference, cm	85.15±8.18	83.06±8.16	80.61±8.96	<.001
Body mass index	24.49±8.88	24.48±3.05	24.17±4.28	0.115
Smoking history, n (%)				0.372
current smoker	8 (4.17)	7 (3.63)	5 (2.58)	
ex-smoker	1 (0.52)	3 (1.55)	0 (0.00)	
non-smoker	183 (95.31)	183 (94.82)	189 (97.42)	
SBP, mmHg	130.17±16.29	130.66±15.61	129.27±17.05	0.336
DBP, mmHg	82.50±10.23	81.14±10.74	80.96±10.51	0.305
FBG, mg/dL	92.46±20.44	92.68±17.26	88.24±9.31	0.002
FBI, µIU/mL	8.4 (6.77–11.12)	7.8 (6.5–10.2)	7.3 (5.9–8.9)	<.001
HOMA-IR	1.95 (1.46–2.58)	1.74 (1.39–2.39)	1.6 (1.23–1.97)	<.001
Total cholesterol, mg/dL	213.07±43.57	214.33±36.50	206.46±40.23	0.075
Triglyceride, mg/dL	129 (95–203.25)	127 (91–168)	99 (74.25–137.75)	<.001
HDL-C, mg/dL	44.5 (38–50)	48 (42–57)	49 (43–59)	<.001
LDL-C, mg/dL	130.44±38.37	128.55±31.34	121.58±31.35	0.027
CIMT, mm	0.88 (0.75–1)	0.84 (0.71–0.96)	0.8 (0.71–0.92)	0.018
% of high CIMT (>0.9 mm), n (%)	89 (46.35)	74 (38.34)	64 (32.99)	0.026*

SBP, systolic blood pressure; DBP, diastolic blood pressure; FBG, fasting blood glucose; FBI, fasting blood insulin;, HOMA-IR, Homeostasis model assessment-insulin resistance; HDL-C, high-density lipoprotein cholesterol; LDL-C, low-density lipoprotein cholesterol; CIMT, carotid intima media thickness.

* p for trend.

Even though there is evidence to suggest an independent anti-atherosclerotic role for adiponectin [11], it remains unclear whether adiponectin is independently associated with atherosclerosis after controlling for conventional cardiovascular risk factors, such as blood pressure, lipid profile, insulin resistance, and waist circumference in each gender. Even if adiponectin levels are associated with atherosclerosis, independently of other cardiovascular risk factors, it is still uncertain whether adiponectin can make an additional contribution to predicting the risk of atherosclerosis, as an adjunct to conventional cardiovascular risk factors.

The aims of this study were: 1) to elucidate the relation of adiponectin to atherosclerosis for each gender specifically in gender-stratified analyses; 2) to determine whether adiponectin is independent of other cardiovascular risk factors; 3) to evaluate whether adiponectin provides additional information regarding the risk of atherosclerosis, beyond that provided by conventional cardiovascular risk factors.

## Methods

### Study Population

The Korean Genomic Rural Cohort (KGRC) study is a part of the Korean Genome and Epidemiology Study (KoGES), an ongoing multicenter cohort study to assess the prevalence, incidence and risk factors for chronic degenerative disorders such as hypertension, diabetes, osteoporosis, and cardiovascular disease [12]. The KoGES study currently comprises a general adult population in 5 geographic areas from November 2005 to March 2011. Within this cohort, data for both serum adiponectin levels and ultrasound images of the carotid artery were available from 1368 participants aged 45–75. Because adiponectin levels can be affected by pharmacological medications, such as peroxisome proliferator-activated receptor (PPAR)-gamma agonists [13], we excluded 335 participants who were currently being treated with pharmacological medications for diabetes, hypertension, or dyslipidemia, as well as those who were under treatment for cancer, based on self-reported questionnaires. Finally, a total of 1033 subject were included in the statistical analyses.

All participants provided written informed consent to their participation, and the study was approved by the Institutional Review Board of Wonju Christian Hospital.

### Measurement of Anthropometric and Metabolic Characteristics

Comprehensive questionnaires were used to collect medical history. Physical examinations were carried out according to standard procedures [14]. Body weight, height, and waist circumference were measured while participants wore light indoor clothing without shoes. Systolic blood pressure (SBP) and diastolic blood pressure (DBP) were measured twice in the right arm using a standard mercury sphygmomanometer (Baumanometer, USA). The lower of the 2 observations of SBP and DBP was used in the current study.

Venous blood samples were drawn from participants in the morning, after they had fasted for 8 hours. Serum concentrations of adiponectin were measured by radioimmunoassay (RIA) (LINCO Research, Inc., St Charles, MO, USA). Fasting blood glucose (FBG) and insulin (FBI) were determined by a glucose oxidize-based assay and double-antibody RIA. Serum concentrations of low-density lipoprotein cholesterol (LDL-C), high-density lipoprotein cholesterol (HDL-C), and triglycerides were determined by enzymatic methods (ADVIA 1650, Bayer, USA). Insulin resistance was calculated using a homeostasis model (HOMA-IR) according to the following equation:

Insulin resistance (HOMA-IR) = fasting blood insulin (µIU/mL)×fasting blood glucose (mg/dL)/405.

### Ultrasound Imaging of Carotid Intima–media Thickness (CIMT)

As described previously [12], a high-resolution B-mode ultrasound system (Vivid 7, General Electric, Horten, Norway) with a 12-MHz transducer can be used to determine the thicknesses of the bilateral carotid intima–media using semi-automated edge-detection software. The minimum, maximum, and mean values of CIMT were calculated from both sides. The maximum CIMT levels from both sides were used as surrogates of atherosclerosis [15].

### Statistical Analysis

All statistical analyses were performed after stratifying for gender. Data in this study are expressed as frequencies with percent, means with standard deviation, or medians with inter-quartile range. The probability of the risk of atherosclerosis was plotted in relation to the adiponectin level. The odds ratios with 95% confidence intervals were calculated with respect to tertile increments in adiponectin level for both genders. The independence of the effects of adiponectin was estimated by multiple logistic regression models, and the additional effect of adiponectin was evaluated as the area under the receiver operating characteristics (ROC) curve. Furthermore, We calculated the net reclassification improvement (NRI) and the integrated discrimination improvement (IDI) to measure the quantifying improvement of correct reclassification and sensitivity according to addition of serum adiponectin in logistic regression model [16]. Values of *p* (two-tailed) less than 0.05 were considered to be statistically significant.

## Results

### Anthropometric and Metabolic Characteristics (Table 1)

Of the 1033 participants, 454 were men and 579 were women. The values of waist circumference, diastolic blood pressure, FBG, and triglycerides in men were higher than those in women. Body mass index, FBI, HOMA-IR, total cholesterol, HDL-C, and LDL-C levels were lower in men than in women.

### Anthropometric and Metabolic Characteristics According to Tertile Increment of Adiponectin (Table 2)

There was an inverse relationship between waist circumference and tertile increment of adiponectin in men. The levels of FBG, FBI, HOMA-IR and triglycerides decreased, whereas HDL-C increased according to the tertile increment of adiponectin in both genders. In addition, LDL-C levels showed an inverse relationship with the tertile increment of adiponectin in women.

The median levels of CIMT and the percentage of high CIMT decreased with the tertile increment of adiponectin in both genders.

### Logistic Regression Plot for Risk of High CIMT According to Adiponectin Level (Figure 1)

**Figure 1:**
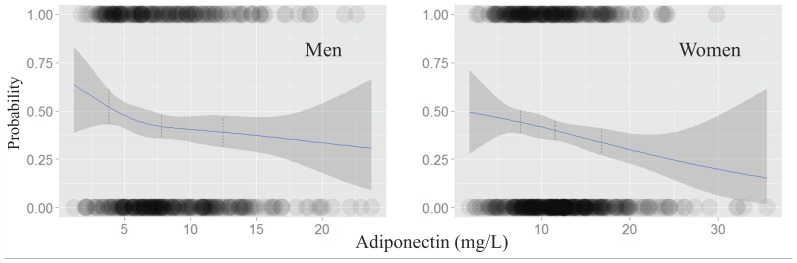
Logistic regression plot for risk of subclinical atherosclerosis (carotid intima media thickness >0.9 mm) according to adiponectin level. The dot lines indicate the median level of adiponectin in each tertile levels (4.71 in 1^st^ tertile, 7.85 in 2^nd^ tertile, 12.52 in 3^rd^ tertile in men; 7.36 in 1^st^ tertile, 11.53 in 2^nd^ tertile, 16.72 in 3^rd^ tertile in women, unit = mg/L).

The risk of a high CIMT decreased in inverse relation to adiponectin levels in both genders. The dotted lines in figure 1 indicate the median level of adiponectin in each tertile level (4.71 mg/L in 1^st^ tertile, 7.85 mg/L in 2^nd^ tertile, 12.52 mg/L in 3^rd^ tertile in men; 7.36 mg/L in 1^st^ tertile, 11.53 mg/L in 2^nd^ tertile, 16.72 mg/L in 3^rd^ tertile in women). The density of the circles indicates the number of samples counted in each range. In each tertile of adiponectin, there were inverse relationships between adiponectin and the risk of high CIMT for both genders.

### Multiple Logistic Regression Models for Risk of High CIMT According to Tertile Increment of Adiponectin (Table 3)

The adiponectin level had an inverse correlation with the risk of high CIMT in both genders, independent of conventional cardiovascular risk factors. When the adiponectin level of the third tertile was compared to that of the first tertile, the OR (95%CI) was 0.45 (0.28–0.74) in men and 0.48 (0.31–0.74) in women, after adjustment for age. Waist circumference and smoking history did not attenuate these associations in either gender. After adjustment for conventional cardiovascular risk factors, such as LDL-C, HDL-C, triglycerides, blood pressure, and HOMA-IR, the OR (95%CI) of the third tertile was 0.42 (0.25–0.72) in men and 0.47 (0.29–0.75) in women.

### The Additional Contribution of Adiponectin to Predicting Risk of High CIMT (Figure 2)

The AUC of the ROC analysis was 0.655 in men and 0.654 in women, based on logistic regression models that included age, waist circumference, smoking history, LDL-C, HDL-C, triglycerides and HOMA-IR, and menopause history in women. The significantly increased, by 0.025 in men and 0.022 in women, when adiponectin was added to the models (AUC in men: 0.655 to 0.680, p = 0.038, AUC in women: 0.654 to 0.676, p = 0.041).

The category-free NRI was 0.32 (95%CI: 0.13–0.50, p<0.001), and the IDI was 0.03 (95%CI: 0.01–0.04, p<0.001) for men. For women, the category-free NRI was 0.18 (95%CI: 0.02–0.34, p = 0.031) and the IDI was 0.003 (95%CI: −0.002–0.008, p = 0.189). Hence, addition of adiponectin to the model reclassified 31% more cases in the correct direction in men and 18% more cases in women, respectively.

## Discussion

This community-based cross-sectional study demonstrated that: 1) there were significant relationships between adiponectin and atherosclerosis in both genders; 2) these associations persisted after adjustment for conventional cardiovascular risk factors in both genders; and 3) adiponectin makes an additional contribution to predicting the risk of atherosclerosis, beyond the information provided by conventional cardiovascular risk factors.

Adiponectin is a kind of circulating adipokine that is associated with endothelial dysfunction [17]. Adiponectin can become attached to the endothelial walls of injured vessels [18] and can control leukocyte–endothelium interactions by up-regulating the expression of endothelial cell adhesion molecules [19]. In addition, adiponectin reduces endothelial nitric oxide synthase (eNOS) activity induced by oxidized low-density lipoprotein or hyperglycemic conditions [20], [21]. These pathophysiologic actions of adiponectin have led many authors to suggest that it may play a protective role against atherosclerosis. Our current results support such a protective effect of adiponectin in both genders.

**Figure 2:**
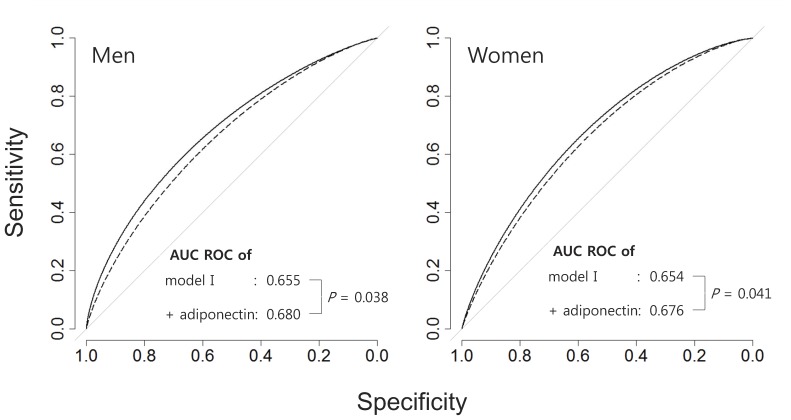
The additional contribution of adiponectin to predicting risk of high CIMT Model I include the age, waist circumference, smoking history, low density lipoprotein cholesterol, high density lipoprotein cholesterol, triglyceride and homeostasis model assessment-insulin resistance (and menopause history in women).

Although there is now a large body of evidence for the protective role of adiponectin against atherosclerosis in both genders, some studies reported that adiponectin did not show an independent association with atherosclerosis [9]. However, these studies did not use gender-specific analyses, despite the fact that levels of serum adiponectin differ greatly between men and women [22]. These hormonal variations may affect the clinical features of atherosclerosis according to gender. In general, serum adiponectin levels are higher in women than those in men, because there is a gender difference in the way it is synthesized and secreted. As with other sex hormones, these gender differences begin at puberty [23]. Specifically, testosterone is positively associated with atherosclerotic risk factors, independently of aging and other risk factors [24]. Furthermore, testosterone inhibits the high-molecular-weight active form of adiponectin [25]. Therefore, careful consideration of the gender effect is a prerequisite in any study of adiponectin and atherosclerosis. In the present study, the adiponectin levels in women were so much higher than those in men that both genders could not be included in a single logistic regression model. Therefore, gender stratification analyses were undertaken to elucidate the exact role of adiponectin in both genders, while the conclusion that adiponectin provides additional evidence for the assessment of atherosclerotic risk was drawn from separate gender-stratified logistic regression models (table 3, figure 2).

**Table 3:** Odds ratio according to adiponectin of subclinical atherosclerosis (carotid intima media thickness >0.9).

			Odds ratio (95% confidence interval)		
			Model I	Model II	Model III
Men ( = 454)	Adiponectin (mg/L)	1^st^ tertile ≤6.29	1 -	1 -	1 -
		2^nd^ tertile >6.29	0.57 (0.36–0.92)	0.55 (0.34–0.88)	0.50 (0.30–0.82)
		3^rd^ tertile >9.90	0.45 (0.28–0.74)	0.44 (0.27–0.73)	0.42 (0.25–0.72)
Women ( = 579)	Adiponectin (mg/L)	1^st^ group ≤9.45	1 -	1 -	1 -
		2^nd^ group >9.45	0.66 (0.43–0.99)	0.59 (0.38–0.91)	0.69 (0.44–1.06)
		3^rd^ group >13.40	0.48 (0.31–0.74)	0.38 (0.24–0.60)	0.47 (0.29–0.75)

Model I: adjusted for age.

Model II: model I+waist circumference and smoking history (and menopause status in women).

Model III: model II+low density lipoprotein cholesterol, high density lipoprotein cholesterol, triglyceride, systolic blood pressure and homeostasis model assessment-insulin resistance.

Adiponectin was inversely correlated with the risk of atherosclerosis, independently of the conventional cardiovascular risk factors. When the adiponectin level in the third tertile was compared to that in the first tertile, the ORs (95%CI) were 0.45 (0.28–0.74) in men and 0.48 (0.31–0.74) in women, after adjustment for age. These associations persisted in both genders after adjustment for conventional cardiovascular risk factors, such as waist circumference, smoking history, LDL-C, HDL-C, triglycerides, blood pressure, and HOMA-IR. Insulin resistance, obesity, and lipid metabolism play an important pathogenetic role in the development of cardiovascular diseases, including atherosclerosis [16], [26]. In the present study, the association between adiponectin and atherosclerosis was not attenuated when HOMA-IR, waist circumference and lipid profiles were taken into account. These results indicate that adiponectin has an adjunctive pathophysiology, different from that of other conventional cardiovascular risk factors.

The total sum of sensitivity and specificity of the various biomarkers was represented by the ROC analysis [27], with a higher AUC indicating a better model for explaining the target outcome. In this study, we demonstrated that adding adiponectin to conventional cardiovascular risk factors increased the AUC (figure 2). The NRI could give clinical information by showing the quantifying improvement by addition of new biomarkers to the previous model. The IDI indicated the increased sensitivity by addition of new biomarkers without sacrificing specificity. Hence, the NRI and the IDI be more sensitive than the AUC in the ROC analysis for indentifying improvements in predictive value [16]. In the current study, addition of adiponectin increased the NRI in both gender, and improved overall sensitivity measured by IDI in men. These results indicate that adiponectin could provide additional clinical information about the risk of atherosclerosis, beyond that provided by conventional cardiovascular risk factors. This study had some limitations. Because of its cross-sectional nature, we were unable to determine a causal relationship between atherosclerosis and adiponectin. In addition, we only examined total adiponectin level rather than the high-molecular-weight and active forms [28]. Therefore, further longitudinal studies using the active form of adiponectin are needed.

In summary, the results of this study support the existence of a significant relationship between adiponectin and atherosclerosis in both genders, independent of conventional cardiovascular risk factors. Furthermore, we demonstrated that adiponectin provides additional information for the assessment of atherosclerotic risk, beyond that provided by the conventional cardiovascular risk factors.
